# Auditory and Visual Cues for Topic Maintenance with Persons Who Exhibit Dementia of Alzheimer's Type

**DOI:** 10.1155/2015/126064

**Published:** 2015-06-10

**Authors:** Amy F. Teten, Paul A. Dagenais, Mary J. Friehe

**Affiliations:** ^1^University of Nebraska Omaha, Omaha, NE 68182, USA; ^2^University of South Alabama, Mobile, AL 36688, USA

## Abstract

This study compared the effectiveness of auditory and visual redirections in facilitating topic coherence for persons with Dementia of Alzheimer's Type (DAT). Five persons with moderate stage DAT engaged in conversation with the first author. Three topics related to activities of daily living, recreational activities, food, and grooming, were broached. Each topic was presented three times to each participant: once as a baseline condition, once with auditory redirection to topic, and once with visual redirection to topic. Transcripts of the interactions were scored for overall coherence. Condition was a significant factor in that the DAT participants exhibited better topic maintenance under visual and auditory conditions as opposed to baseline. In general, the performance of the participants was not affected by the topic, except for significantly higher overall coherence ratings for the visually redirected interactions dealing with the topic of food.

## 1. Introduction

Healthcare providers rank individuals with dementia as the third most common disorder served on their caseloads [1]. It is well known that persons with Dementia of Alzheimer's Type (DAT) experience a progressive decline in memory and intellect. These deficits eventually lead to problematic language and discourse in which more discrete skills such as syntax and phonology are left relatively preserved [2], while deficits in the conceptual, semantic, and pragmatic aspects of language are apparent [3]. Such deficits result in numerous difficulties for caregivers when attempting to communicate with persons with DAT. Due to the memory, attentional, and pragmatic deficits of persons with DAT, the caregiver is often left to compensate for the impaired person's inability to stay focused on one topic. Functionally, it is difficult to sustain conversational interactions that deal with important daily topics. Therefore, it is important to investigate indirect approaches utilizing the caregiver as a reasonable alternative to direct cognitive approaches with the impaired person.

While healthy elderly adults have been found to be able to confine themselves to one topic when instructed [4], persons with DAT exhibit poor topic maintenance when engaging in discourse [5, 6]. A few of the problems exhibited by persons with DAT when engaging in discourse include

- poor topic maintenance and reduced inclusion of core elements required by the topic [7–9],
- reference errors, sentence fragments, and difficulty formulating and remembering the content of sentences [2, 5, 10],
- circumlocution, perseveration, and revisions [7, 11].

Another discourse variable which has been studied in DAT includes topic relevance. Individuals with DAT have been found to produce a high proportion of irrelevant information and slightly more redundant information than healthy elderly controls [12]. Even minimally impaired patients showed a significant decline in the number of information units produced on a discourse task [13–15].

Other literature has attempted to measure the coherence of DAT discourse. Persons with DAT were found to be significantly impaired with respect to global coherence (i.e., the ability to maintain overall topic unity) relative to normal controls [3, 16]. In related studies, researchers [17, 18] found a deficit in coherence along with disrupted cohesion (i.e., the use of devices such as coreference to link one sentence to the next in an effort to maintain coherence). Specific to moderate to severe DAT, members of this population exhibited poorer topic introduction skills and unexpected topic shifts [19, 20].

Given the deficient coherence in DAT discourse, need for discourse repair is apparent. Various authors [9, 21, 22] have discovered that, in most situations where repair is needed in interactions with persons with DAT, caregivers take the initiative in the repair process. Caregivers have been found to utilize a variety of strategies for facilitating repair:

- Paraphrasing relevant ideas [22].
- Asking yes/no questions to structure the repair [23].
- Restating the topic, repeating ideas, or providing missing information [24].
- Removing embedded clauses, using one idea per utterance, and using gestures to supplement caregiver verbalizations [25, 26].

It appears as if the effectiveness of caregiver repair strategies is somewhat dependent upon severity of DAT, with more success occurring during earlier stages of the disease. Various authors [25, 27] have found that caregivers of persons with early DAT were better judges at determining the effectiveness of specific repair strategies. Caregivers of persons with later-stage DAT often exhibited mismatches in terms of what they reported as useful versus what actually resolved communication breakdown as determined by the investigators. The authors reported that conversational partners of persons with early DAT were able to use a wider variety of repair strategies while fewer strategies were attempted by those caregivers of later-stage DAT participants.

Gentry and Fisher [28] investigated the use of indirect versus direct repair strategies initiated by the conversational partner of participants with DAT. Indirect repairs were considered to be strategies such as paraphrasing the ideas of the person with DAT. Direct repair strategies involved corrective feedback. Indirect repair strategies were found to yield greater coherence in DAT communication attempts. In addition, these indirect repairs resulted in the DAT participants generating more words, shifting topics less, and engaging in communication for longer durations compared to direct repair strategies.

Various visual and auditory cues have been used by investigators to improve communication. For instance, Bourgeois [29] attempted to enhance the conversational skills of persons with DAT by implementing the use of a prosthetic memory aid. Participants were trained to increase factual information on three different topics by referring to a set of pictures and sentences kept in a wallet. The author found that use of the aid doubled to tripled the factual statements used by DAT participants after training was completed. Other researchers [10] used auditory cues to prompt persons with mild to moderate DAT to verbalize more specific referential information regarding pictured stimuli. When receiving only one auditory cue, DAT participants were able to add the desired information only one-third of the time. The authors determined that DAT participants required a higher number of cues and more explicit auditory cues in order to elicit desired information.

Fried-Oken et al. [30] compared visual-only augmentative and alternative communication (AAC) support for conversation versus visual-plus-auditory AAC support when persons with moderate DAT interacted with conversational partners about preferred topics. Visual-only support took the form of sixteen pictures with corresponding printed word labels while visual-plus-auditory support combined the pictures plus labels with digitized speech output when the participant selected the picture on a speech-generating device. The authors found that visual-only support yielded the best results in that persons with DAT generated more words per utterance, more total utterances, and more topic elaborations and/or initiations. In contrast, the addition of the digitized speech output was thought to “depress conversational performance and distract participants” with DAT [30].

A revision to the above study included a training component whereby participants with DAT received spaced retrieval training in order to familiarize them with the picture layout of the low-tech AAC visual support prior to data collection. The authors found that the addition of the training component plus the visual-only AAC support increased use of target words in conversation [31].

Previous research has been comprehensive in describing the characteristics of discourse in persons with DAT. A few studies have focused on topic relevance and repair with most studies indicating the burden of repair falls to the nonimpaired conversational partner. Mixed results have been found in relation to what types of caregiver cues (e.g., visual versus auditory) best support communication, and, to our knowledge, no studies have looked directly at the effects of caregiver cues on topic maintenance/coherence regarding topics related to activities of daily living. Given the focus on indirect techniques to improve communication for persons with DAT found in the literature, a system of caregiver cueing should be explored to determine its effects on improving in-the-moment conversational interactions between persons with DAT and their caregivers.

Therefore, it was proposed that studying ways to manipulate how individuals converse with persons with DAT might yield changes in the conversational output of persons with DAT. In contrast to earlier studies, the present study put virtually no restraints on how the persons with DAT chose to interact. Instead, it looked to improve topic maintenance in interactions with persons with DAT by changing the way a conversational partner interacted with them. Specifically, the following research questions were posed:

1. Can manipulation of a conversational partner's role in interactions with persons with DAT effect a positive change in DAT participants' topic maintenance skills?
2. Will a visual redirection to topic improve the topic maintenance skills of persons with DAT?
3. Will an auditory redirection to topic improve the topic maintenance skills of persons with DAT?
4. Will there be a difference in the effectiveness of visual versus auditory redirections?

## 2. Methodology

### 2.1. Participants

#### 2.1.1. Adults Used to Pilot Research Task

Prior to administering the research task, five healthy elderly (HE) adults were recruited from an assisted living facility to ensure that age- and education-matched peers would be able to exhibit the expected topic control required of the task [4]. They were matched to participants with DAT according to age (±5 years), education (±2 years), and gender. Members of the pilot group were screened for cognitive impairment by the primary investigator using the Mini-Mental State Examination (MMSE) [32]. Any score higher than 23/30 was considered a passing score. These adults also determined which photos would be utilized in the visual condition of the research task. In general, pilot data showed these participants required few redirections to topic and maintained greater than 80% topic coherence as judged by the Glosser and Deser [3] topic coherence scale described in the procedures below.

#### 2.1.2. DAT Participants

Five adults with language disorder secondary to DAT participated in this study. They were selected from a memory-loss unit at the same assisted living facility as the adults who piloted the research task. A neurologist had provided a diagnosis of probable DAT for all participants. Demographic information regarding participant status is summarized in Table 1.

Each participant was rated using the Global Deterioration Scale (GDS) [33]. The GDS is used to rate the severity of dementia on a 7-point scale where a rating of 1 corresponds to “no cognitive decline” and a rating of 7 refers to persons with “very severe cognitive decline.” Persons at stages between 2 and 4 are considered to be in “confusional” states while persons at stages 5 through 7 are considered to have dementia.

Persons rated at levels 5 and 6 on the GDS were targeted for the study. Azuma and Bayles [5] stated that persons at these levels produce less spontaneous language, have a limited vocabulary, and exhibit problems with cohesion between sentences. At the same time, they still have intact syntax. These persons are rated as having a moderately severe to severe cognitive impairment. Three persons highly involved in the day-to-day care of the facility's residents provided participant ratings. An average score was computed for each participant. To be included in this study, a participant had to receive an average score between 4.67 and 6.33. This range incorporated the 5-6 characteristics described by Azuma and Bayles [5] without omitting from consideration someone who received two 5s and a 4 or two 6s and a 7 by the raters.

In addition, all participants' language skills were measured by the primary investigator who administered the Arizona Battery of Communication Disorders of Dementia (ABCD) [34]. All DAT participants exhibited language difficulties secondary to cognitive impairment as evidenced by overall scores near published norms for persons with mild AD (overall score *M* = 18.12) and moderate DAT (overall score *M* = 10.15. See Table 1).

Participants for either group were excluded if they had any prior history of psychological, neurological, language, or speech disorder. They were also excluded if any history of drug/alcohol abuse was reported. All participants had vision and hearing, corrected if needed, adequate to perform the task. This was confirmed by participant self-report and by staff from the assisted living community.

### 2.2. Procedures

Consent to participate was obtained from each participant's caregiver. Then, a detailed case history was obtained for each. The DAT participants were asked to give verbal consent at the beginning of each session. Also, the day-to-day assent of the participant was considered withdrawn if the primary investigator observed continued signs of agitation on the part of the participant. Regarding assent, one participant was too agitated to begin the procedures on one day. The investigator came back another day at which time the participant was able to tolerate the procedures. The approximately 20 minutes needed for the daily conversations was short enough that no participants became agitated once they entered the room where conversations took place. Testing took place in a quiet environment familiar to the participant. The participant and the primary investigator sat in close proximity, enabling them to see each other and the stimulus materials used in the visual condition. All procedures were audio- and video-recorded across three days, as described below.

#### 2.2.1. Baseline Procedures

Baseline data was obtained from three five-minute interactions with each participant. One baseline conversation took place for each of the three days. Baseline conversations were paired with one auditory-redirected conversation and one visually redirected conversation for each day. In this manner, all three topics and all three conditions were presented only once each day to reduce potential perseveration (see general procedures below). During each interaction, three different topics that represent commonly occurring daily living activities were presented (food, grooming, and activities). Enabling persons with DAT to better communicate regarding these topics would be highly ecologically valid. Also, the authors felt that improving communication in these situations would result in better quality of life for the participants.

The primary investigator opened each conversation by using an open-ended question. For the activities topic, the participant was asked, “What would you like to do today?” For the food condition, participants were asked, “What would you like to eat today?” And for the grooming condition, participants were asked, “What do you do to get ready in the morning?” These prompts were chosen due to there being normally occurring efforts to initiate communication around daily living events. The primary investigator's role in these interactions was limited to providing minimal turns. Minimal turns were defined as any short utterance that provided no additional content to the conversation but simply turned the conversation back to the participant. The primary investigator only deviated from this procedure if the conversation was at a standstill. For the baseline condition, a standstill was defined as the participant not taking a conversational turn with corresponding silence for 10 seconds. At a standstill, the primary investigator asked, “Is there anything else you'd like to tell me?” The topic was not restated.

#### 2.2.2. Auditory Condition Procedures

In the auditory condition, the same three topics were introduced in the same format as the baseline condition. Each interaction was set to last for five minutes. Progress in the interaction was evaluated every 30 seconds as timed with a stopwatch. If the participant was successful at maintaining the topic, the primary investigator only took minimal turns. If the participant was off topic, an auditory cue (e.g., “Remember, we're talking about…now.”) was interjected by the primary investigator. Once an auditory cue was given, the primary investigator returned to taking minimal turns. Thirty seconds later, progress was again evaluated with the primary investigator making the same forced-choice decision, using only minimal turns if the interaction remained on topic or providing the same auditory cue if the discourse had deviated from the topic.

#### 2.2.3. Visual Condition Procedures

Photographs corresponding to the three topic areas (i.e., food, grooming, and activities) were placed in front of the primary investigator and the participant for the visual condition. Photographs chosen for the study were those that could be correctly named by the healthy elderly adults in one try. The pool of photographs relating to each topic was then narrowed down by asking the healthy elderly adults to choose the six photographs per topic that they felt would best help them answer the questions to be presented (i.e., “What would you like to eat today?”). (See the Appendix for a list of photographs used for each topic.)

One card containing six photographs organized in two rows of three and pertaining to a specific topic was used for each five-minute interaction. The primary investigator directed the participant's attention to the photos and opened each topic in the same manner as for the other two conditions. At the end of every 30 seconds, progress was evaluated. If the participant was successfully maintaining the topic, the primary investigator took minimal turns. If the participant was off topic, the primary investigator called the participant's attention to a specific photograph without using the word for the target concept depicted in the picture by stating, “What about this?” For the succeeding thirty seconds, the primary investigator took minimal turns. Progress was evaluated in this manner every thirty seconds. (Note that although there was an auditory component to the visual condition, it will continue to be referred to as the visual condition with more commentary in the Discussion.)

#### 2.2.4. General Procedures

Conversations took place in the midmorning or midafternoon as per facility wishes. The primary investigator aimed for times that would be approximately two hours after the most recent meal as well as two hours prior to the next meal to minimize potential effects of hunger on the conversations about food.

The order in which each subject participated in visual versus auditory versus baseline conditions was pseudorandomized with the following constraints: persons with DAT were only allowed to participate in three interactions daily in an attempt to reduce possible perseveration. Therefore, each of the three topics was represented daily. Therefore, for both conditions and topic, all three conditions and all three topics had to be presented daily (e.g., a person might engage in the visually redirected grooming topic, followed by the baseline activities topic, followed by the auditorily redirected food topic. In this manner, no condition or topic was repeated in one day). With three conditions (A, B, and C) and three topics (1, 2, and 3), nine potential condition × topic pairs existed (A1, A2, A3, B1, etc.). The order in which these possible pairs were presented was then randomly assigned. The primary investigator administered all tasks.

Picture stimuli for the visual condition were rotated between interactions with participants to negate any possible ordering effects of picture presentation. Eye contact and tactile stimulation were maintained in all conditions, as tolerated and appropriate, to increase the likelihood of the participant's continued attention to the task. Tactile stimulation consisted of squeezing each participant's hand when entering the testing environment in an attempt to orient the participant's attention to the primary investigator. This was accompanied by the statement “Thank you for talking with me today.”

All conditions were audio- and video-recorded. A simple nonverbal distractor task (i.e., a letter cancellation task) was presented between topics to decrease the likelihood of perseveration from interaction to interaction. A total of 3 days were needed to complete the 9 experimental interactions (3 topics × 3 conditions ÷ 3 conversations per day). At the end of each five-minute conversation, the primary investigator thanked the participant for sharing his/her ideas about the topics to signal the end of the conversation. If conversations went slightly beyond five minutes, only the first five minutes were transcribed to be analyzed.

#### 2.2.5. Scoring

Videotapes of the interactions were reviewed, and typed transcripts of the first five minutes of each interaction were completed by both the first author and a graduate student in a speech-language pathology program who was trained by the first author. In a sample of 25% of transcripts completed by both transcribers, there was greater than 95% agreement in all aspects of transcription.

Five minutes was chosen as the target for the conversations so that they should be sufficiently long to ensure that the cuing system would be used. The authors felt that a shorter interaction would not provide enough 30-second intervals at which cues might be useful. The five-minute timing of interactions will be further deliberated in the Discussion. Gestures that were noticeable on video were included in the transcripts in brackets. Gestures, by themselves, were not scored. However, gestures that added context to a participant's spoken utterance were scored with that utterance.

The primary investigator first scored all transcripts while viewing the videotape. A second rater, thoroughly trained in the procedures, viewed the tapes of approximately 25% of the interactions and scored them for reliability purposes. Transcripts to be scored by the second rater were randomly selected.

Each utterance was coded on a 1–5 global coherence scale adapted from Glosser and Deser [3]. Scores (with examples related to the food topic) were as follows:

1. A score of 1 indicated that the participant's utterance did not relate to the initially stated topic at all (e.g., “I liked being on the boat”).
2. A score of 2 was assigned if the utterance contained a vague reference to topic, perhaps with no specific referent (e.g., “That stuff I like is delicious”).
3. A score of 3 corresponded to an on-topic statement not related to the present day (e.g., “My mother used to make the best ham-bone soup”).
4. A score of 4 was assigned if an on-topic statement diverged slightly from its relevance to the present day (e.g., “I really like that salad they have here,” unclear if participant wants it today).
5. A score of 5 was awarded if the participant's utterance was logically related to all elements of the topic (e.g., “I'd really like that soup today”). Therefore, when the original statement of topic was “What would you like to eat today?” the participant had to address the time factor appropriately (today) as well as the general subject matter (favorite foods) in order to receive a score of 5. The authors believed that the factor of time was relevant to determining the participants' current likes/dislikes to participate in conversations about ADLs efficiently.

Any utterance on which the primary investigator's and second rater's assigned scores differed by more than 1 point was rectified by a consensus of both scorers. In cases where scores differed by only one point, the primary investigator's score was used. Interjudge reliability scores were computed by the following formula:

(1) $$\begin{array}{c} \frac{\text{total number of agreements}}{\text{total number of utterances}}. \end{array}$$

Reliability for the two judges was found to be 84% prior to rectifying scores.

An overall coherence score was calculated from each transcript. Overall coherence was defined as the ratio of the total number of points awarded to the total number of possible points for that interaction. This score represented the topic maintenance abilities of each participant for each entire interaction regardless of the presence (visual and auditory conditions) or absence (baseline) of primary investigator-directed cues to topic.

### 2.3. Statistical Analysis

Overall coherence data was analyzed by two-way ANOVA (condition × topic). Subsequent post hoc testing was completed as needed. Subsequent to the study, a power analysis [35] was performed to determine whether the number of participants used in this study was adequate for determining differences between conditions. To replicate this study and be 90% sure to replicate the finding that conversations aided by auditory and visual redirections were significantly more coherent than baseline conversations, one participant would have to be studied (effect size *d* = 5.29). To replicate the condition × topic interaction whereby coherence for the visual redirection/food topic combination was significantly higher than other visually redirected topics and the auditory redirection/activities topic combination yielded better coherence than other topics under auditory redirection, four participants would be needed (effect size *d* = 1.75). Therefore, the number of participants (five) was sufficient to support the significance of the findings.

## 3. Results

Piloting with healthy elderly (HE) adults revealed that, using the stated procedures, all healthy elderly pilot participants achieved near-ceiling-level topic coherence abilities for the experimental task as expected. Topic coherence for these HE participants averaged 0.82 (or roughly 4.1 points out of 5 for each utterance) for all topics. This is consistent with data reported by Glosser and Deser [36] who reported good cohesion between contiguous utterances for HE participants. However, their study found that HE participants were less likely to maintain the general topic over the course of an informal interview than middle-aged participants.

**Figure 1:**
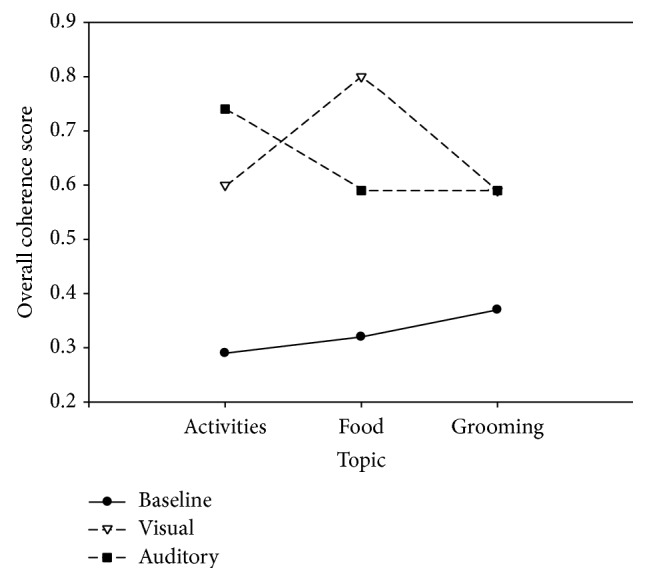
Mean overall coherence scores for DAT participants across baseline, auditory, and visual conditions as well as the topics of activities, food, and grooming.

Mean overall coherence scores across topics and conditions for DAT participants are found in Table 2 and Figure 1. Topics did not seem to affect overall coherence considerably. However, overall coherence did appear to be affected by condition in that persons with DAT performed better in the auditory and visual conditions as opposed to baseline.

Results of two-way ANOVA (condition × topic) found significant differences for condition (*F*(2,16) = 25.127, *p* < 0.05), with no significant difference for topic (*F*(2,16) = 0.253, *p* > 0.05). There was one significant two-way interaction. Condition × topic significantly interacted (*F*(4,32) = 3.315, *p* < 0.05).

To further explore the performance of the DAT participants, six one-way ANOVAs were completed: one for each condition and one for each topic. Overall coherence scores did not differ significantly in the baseline condition across the three topics (*F*(2,8) = 0.626, *p* > 0.05). The auditory condition did produce a statistically significant difference in DAT performance (*F*(2,8) = 5.014, *p* < 0.05). Newman-Keuls post hoc analysis revealed that auditory redirections resulted in overall coherence scores to be highest for the topic of activities (*M* = 0.74, or roughly 3.95 points per utterance). Scores for this topic were significantly higher (*p* < 0.05) than the food and grooming topics (*M* = 0.59 and 0.59, or roughly 2.95 points per utterance, resp.) which did not differ from each other. For the visual condition, a significant difference was also found (*F*(2,8) = 6.174, *p* < 0.05). Post hoc testing showed that, under visual redirection, DAT participants performed significantly better (*p* < 0.05) for the food topic (*M* = 0.79, or roughly 3.95 points per utterance) than they did for either activities (*M* = 0.59, or roughly 2.95 points per utterance) or grooming (*M* = 0.58, or roughly 2.9 points per utterance). The latter two topics were not statistically different.

For the topic of activities, significance was attained (*F*(2,8) = 25.923, *p* < 0.01), with the auditory and visual conditions yielding significantly higher overall coherence scores (*p* < 0.05) than the baseline procedure. The topic of food yielded a statistically significant difference (*F*(2,8) = 35.712, *p* < 0.01) between all three conditions with overall coherence scores for the visual condition being greater than for the auditory condition (*p* < 0.05) which was greater than the baseline condition (*p* < 0.05). A significant difference also was found for the topic of grooming (*F*(2,8) = 6.778, *p* < 0.05). Baseline scores were found to be significantly lower (*p* < 0.05) than the scores obtained during the auditory and visual conditions, which did not differ.

As an estimate of effect sizes for differences found between baseline and the redirected conditions, a Nonoverlap of All Pairs (NAP) test was completed [37]. As there was no overlap between any baseline data point and any redirected conversation data point for any topic, NAP was calculated to be 100%. This indicates no overlap between coherence ratings on any redirected conversation as compared to baseline conversations.

## 4. Discussion

The purpose of this study was to determine the effectiveness of auditory and visual redirection to topic on the connected discourse of persons with Dementia of Alzheimer's Type (DAT). Specifically, the redirections were intended to yield better in-the-moment topic maintenance for topics of daily significance. This research contributes to the literature by exploring the effects of caregiver cues on topic maintenance/coherence regarding topics related to activities of daily living. In addition, the research found that both the auditory and the visual redirections given by the caregiver were successful in improving the DAT participants' topic coherence. The measure of topic maintenance used for this study was taken from Glosser and Deser [3]. In that system, any score under 3 (of a possible 5) would indicate that an utterance had very poor relatedness to the overall topic. As such, any proportion of total awarded points to total possible points less than 3/5 (0.6) would indicate that more irrelevant information was present than relevant information while the lowest possible ratio would be 1/5 (0.2).

The first research question addressed how changes in a conversational partner's role would impact the topic coherence of persons with DAT. Indeed, results showed that DAT participants can be successfully redirected to a topic, resulting in better overall coherence (the relatedness of every utterance in an interaction to the topic) when either type of redirection was present. These findings are consistent with numerous authors who have reported that communication repair, initiated by the caregiver of a person with DAT, is successful [23, 28].

DAT participants in this study achieved a mean overall coherence score during baseline conditions of 0.32 (or roughly 1.6 points per utterance). This confirmed findings indicating that high proportions of irrelevant information were present in the discourse of persons with DAT [12]. In contrast, when redirection from the conversational partner was present, average overall coherence nearly doubled to 0.65 (or roughly 3.25 points per utterance), thus illustrating the effectiveness of simple, indirect repair strategies initiated by the caregiver.

The secondary goal of the study was to determine whether visual and/or auditory redirections to topic improved topic coherence for persons with DAT. Further, the researchers queried whether there would be a difference in effectiveness between the two modalities of cueing. Results showed that both auditory and visual redirections were equally effective in redirecting participants to a particular topic. Specific to auditory redirections, the present study found that auditory redirections increased topic coherence significantly above baseline levels. This finding is consistent with research which found that the most effective form of repair initiated by the conversational partners was in an auditory format (e.g., by paraphrasing relevant ideas, restating the topic, or repeating ideas) [22, 24]. The current study plus other authors [22, 24] found that redirections by conversational partners facilitated continued interaction. However, the present study showed auditory cues were effective in all conversations whereas other research found that auditory cues were only effective in one-third of interactions [10].

The current results regarding visual redirections also showed improved topic coherence as compared to baseline conversations. These results support the findings of Fried-Oken et al. [30] who determined that visual-only picture support for persons with moderate DAT yielded more topic elaborations and/or initiations. Likewise, visual supports in the form of a prosthetic memory aid also improved on-topic statements [29].

Of interest in this study, two condition × topic pairs were found to significantly improve DAT participants' topic maintenance skills above and beyond the general improvement noted. First, the visually redirected topic of food yielded significantly higher scores for persons with DAT than any other topic under the visual condition. It is possible that persons viewing the pictures had a somewhat visceral reaction, creating more robust discourse and enthusiasm for maintaining topic. DAT performance approached the performance of the pilot HE group for this specific communicative interaction. Also, the auditorily redirected topic of recreational activities brought about significantly higher performance for DAT participants than any other topic under the auditory condition. Persons with DAT may have viewed the primary investigator as a potential partner for engaging in the day's activities, thus yielding more relevant comments. The performance of the pilot HE participants in this study was unremarkable as their performance approached a ceiling effect.

Unlike previous research, the present study attempted to make the utterances of persons with DAT more relevant during discussions about topics related to activities of daily living. Participants were provided with auditory and visual redirections to topics. Redirections were used as intervention in this study based on the findings of Watson et al. [22] who reported that the conversational partners of persons with DAT most often carry the burden of conversational repair. DAT participants appeared to be most successful at topic maintenance during discussions about food with visual redirection and discussions regarding activities during the auditory condition.

The present study showed that structured partner-initiated repair, in the form of auditory or visual redirections, was beneficial to the topic maintenance skills of persons with DAT (see Figure 1). These findings may have practical implications for caregivers of persons with DAT in nursing homes, at assisted living facilities, or at home. The effect of visual redirection on topic maintenance skills during conversations regarding food appeared to be particularly robust (see Figure 1). Creating a set of picture menus could be a cost-efficient way to help persons with DAT express their likes and dislikes while also allowing them to assist in the decision-making process in a more time-efficient and coherent manner.

Further research in this area should be explored. With a larger sample size, some effects that were not present in this study might be found. Adding participants in more mild and severe stages of DAT might also yield some cross-sectional data about the effectiveness of redirection on topic coherence in multiple stages of the disease. Similarly, getting information on coherence abilities from DAT participants who have different levels of education might be of interest. Although one participant in this study had a relatively low level of education, her scores on the ABCD matched closely with the other participants. As a result, we felt that it was appropriate to include her in our analysis given her similar cognitive-linguistic skills. For all DAT participants, a measure of day-to-day variation in performance should be investigated. Finally, a measure of caregiver satisfaction with the redirection system would be of use.

Methods for this type of research may need to be revised. In retrospect, a five-minute time limit on interactions was thought to be excessive. Some of the healthy elderly pilot participants reported finding it very difficult to sustain such limited topics for five minutes. Many of the healthy elderly participants would have required no redirection if the interaction was shorter. In the future, cues should be revised such that the visual redirection is purely visual. In the current study, the visual redirection used also had an auditory component. One cannot be certain that the effect of the “visual” condition was due to the presence of the pictures alone. It would be interesting to see the effects of a pure auditory cue, a pure visual cue, and an auditory-visual cue. Also, there were six choices available as visual cues in the visual condition (i.e., six different pictures). Conversely, there was only one standard cue (e.g., “Remember we're talking about what you'd like to eat today”) for the auditory condition. Although the auditory cue was differentially effective at redirecting conversations about activities, it would be important to know if a cueing system with six choices, such as the visual cueing system, would yield different results. In addition, limited statistics were completed to measure effect sizes of the available data. However, visual inspection of the data and use of Nonoverlap of All Pairs (NAP) analysis indicate strong effectiveness of visual and auditory redirections on topic coherence when compared to baseline conversations.

Cues were chosen based on items that healthy elderly individuals felt were most representative in answering the topics broached so that a standardized set of cues could be used. Future research might look at how person-centered choices of cues for each individual with DAT might change the results. In addition, the cues in the present study were designed more as direct repair strategies [28]. Since Gentry and Fisher found that indirect repair strategies were more effective than direct repairs, it would be interesting to design a cue type to replicate their use of indirect repairs. Also, although the coherence rating scale used is subjective, it was felt to be the most empirical in the literature. Future research might aim to modify this scale or create a new, less subjective scale.

## 5. Conclusions

This study added to the literature regarding caregiver role in repairing discourse with persons who have DAT. A systematic set of cues (redirections) was successful in eliciting discourse that remained at a high level of topic coherence. Both auditory and visual redirections yielded relevant information from persons with DAT in relation to topics necessary for daily living. This information could be used to further explore the effectiveness of simple, indirect redirections to topics of daily significance with larger and more diverse groups of persons with DAT. In addition, long-term use of the cueing system could be explored to determine whether any gains are made once the interactions are made more procedural and therefore routine.

## Figures and Tables

**Table 1:** Demographic data and testing data for DAT participants. Descriptors include gender, age (in years.months), years of education (Yrs. Ed.), scores on the Global Deterioration Scale (GDS), and scores on the *Arizona Battery for Communication Disorders of Dementia (ABCD)*.

Participants with DAT					
Initials	Gender	Age	Years of education	GDS	ABCD
KK	F	82.6	14	5.66	7.75
RD	F	79.9	14	5.66	10.25
OR	F	86.6	8	5.33	11.25
MC	F	79.6	14	5	11.95
WW	M	81.2	12	5.2	18.85

**Table 2:** Means and standard deviations of overall coherence scores according to condition (baseline, auditory, or visual) and topic (activities, food, or grooming).

Condition	Activities	Food	Grooming
Baseline	0.29 (0.06)	0.32 (0.09)	0.37 (0.28)
Auditory	0.74 (0.14)	0.59 (0.21)	0.59 (0.19)
Visual	0.59 (0.17)	0.79 (0.13)	0.58 (0.22)

## Appendix

*Photographs for Activities Topic*. Deck of cards, flower bed, Bible, newspaper, television, and telephone were used.

*Photographs for Food Topic*. Corn, grapes, pork chop, chicken leg, glass of milk, and chocolate chip cookie were used.

*Photographs for Grooming Topic*. Washcloths, pills in a clear plastic cup, toilet, toilet paper, comb, and toothbrush were used.
